# The 100 most‐cited articles about the role of neurovascular unit in stroke 2001–2020: A bibliometric analysis

**DOI:** 10.1111/cns.13636

**Published:** 2021-03-25

**Authors:** Lv Xie, Bingwei Lu, Yezhi Ma, Jiemin Yin, Xiaozhu Zhai, Chen Chen, Wanqing Xie, Yueman Zhang, Li Zheng, Peiying Li

**Affiliations:** ^1^ Department of Anesthesiology State Key Laboratory of Oncogenes and Related Genes Shanghai Cancer Institute Renji Hospital School of Medicine Shanghai Jiaotong University Shanghai China

**Keywords:** bibliometric analysis, blood‐brain barrier, neuroprotection, neurovascular unit, stroke

## Abstract

**Background:**

The neurovascular unit (NVU) is emerging as a potential therapeutic target in neurological conditions, such as stroke, brain injury, Alzheimer's disease, and Parkinson's disease; meanwhile, stroke is the second leading cause of death globally. The purpose of the study is to analyze the most influential articles, authors, countries, and topics in the role of NVU in stroke.

**Methods:**

The Web of Science (WoS) database was used for bibliometric analysis using the search terms “Stroke” and “Neurovascular unit” on January 1st, 2021. Data were extracted from the WoS database to identify collaborations between authors, countries, organizations, and keywords using VOSviewer (1.6.16 mac). Two bibliometric indicators, the activity index (AI) and category normalized citation impact (CNCI), were computed. The keywords of bursts were also identified by CiteSpace.

**Results:**

A total of 770 articles were analyzed by VOSviewer. AIs and CNCIs were computed of the eighteen countries according to VOSviewer co‐authorship analysis results. The majority of authors mainly came from the United States and Japan. Romania, Hungary, and Poland have emerged as rising‐star countries. In the 100 most‐cited articles, the number of citations ranged from 1873 to 69, with a total of 15,758 citations. Most articles were published in 2011 and 2012 (*n* = 13 each), followed by 2009 (*n* = 11) and 2013, 2014, and 2015 (*n* = 8 each). *Stroke* and *Journal of Cerebral Blood Flow and Metabolism* were the two top journals. EH Lo from Harvard University/ Massachusetts General Hospital was the top first author and corresponding author. Harvard University/Massachusetts General Hospital was the most productive affiliated institution with 15 publications.

**Conclusion:**

There has been growing attention and efforts made in the field of stroke and NVU. The merit of the above findings may help to shape the research policy in ischemic stroke both at the country and institutional level.

## Graphical abstract

We performed VOSviewer analysis of collaborations between authors and countries and found two different clusters of authors mainly from USA and Japan had a broad collaboration with authors from other countries. The Lo EH research group and the Abe K research group showed most extensive collaboration with other research groups.

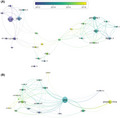

## 1 INTRODUCTION

Stroke is the second leading cause of death globally^1^ and has become the fifth leading cause of death in the United States, which leaves an enormous burden on patients' families and the society at large.^2^ The incidence and mortality of stroke decreased in high‐income countries between 1990 and 2010. However, no significant change has been seen in low‐ and middle‐income countries.^1^, ^3^, ^4^ Stroke can be divided into ischemic stroke and hemorrhagic stroke, and our studies include both stroke types. Nowadays, with standard treatments such as tissue plasminogen activator (tPA) or thrombolytic therapy and mechanical thrombectomy,^5^ more ischemic stroke patients survive the stroke but with complications such as hemorrhagic transformation, cerebral edema, epilepsy, and pneumonia.^6^, ^7^, ^8^, ^9^ Microvascular reperfusion injury following mechanical thrombectomy is also an urgent problem to be solved. Thrombolytic therapy requires a narrow time window and is only used in about 7% of patients after an acute ischemic stroke in the United States.^10^ One novel thrombolytic, desmoteplase, has been reported to have failed the Phase IIb/III trial with no significant improvement in functional outcome at 3 months,^11^ but other thrombolytics and reperfusion agents, such as tenecteplase and stachybotrys microspore triprenyl phenol‐7 (SMTP‐7), remain in development.^12^ Basic studies and clinical trials may uncover next‐generation thrombolytic drugs or agents that could reduce the risk of thrombolysis‐associated hemorrhagic transformation that may allow an extension of the current narrow therapeutic window and reduce the risk of catastrophic brain hemorrhage after stroke. The emergence of the neurovascular unit (NVU) concept provides a new foothold for that research in this regard.

The concept of the NVU, which is comprised of neurons, astrocytes, pericytes, microglia, endothelial cells, and vascular smooth muscle cells (VSMCs), was formalized at the 2001 Stroke Progress Review Group meeting of the National Institute of Neurological Disorders and Stroke (https://www.ninds.nih.gov/About‐NINDS/Strategic‐Plans‐Evaluations/Strategic‐Plans/Stroke‐Progress‐Review‐Group) and demonstrated that components of the NVU in the central nervous system communicated dynamically with each other and maintained hemostasis of the brain.^13^ The interaction and signaling between different components of the NVU are very complicated, and latest advances have shown that the NVU might regulate cerebral blood flow and maintain brain homeostasis through neurovascular coupling or vasculoneuronal coupling.^13^, ^14^, ^15^ Meanwhile, it was recently suggested that astrocytes or microglia, which have multiple phenotypes, have controversial effects on the regulation of neuroinflammation or neurovascular coupling and require further investigation.^16^, ^17^, ^18^, ^19^ Due to the complexity of the NVU, there has been an increasing number of studies on the NVU in stroke. However, currently, none of these studies have translated into new stroke treatments.^20^, ^21^, ^22^, ^23^, ^24^ Considerable efforts have been devoted to finding effective therapeutic targets and pathways in the NVU for not only ischemic stroke, but also a number of other neurological diseases, such as Alzheimer's disease and Parkinson's disease.

The latest progress and the most vital contributions in stroke and the NVU can be found in the surging number of publications in the field of NVU, including reviews, basic studies and clinical trials. However, there is no bibliometric analysis of stroke and NVU. It is of great importance to look into the distribution of articles and authors in this field, which would give readers a vivid overview of the most important works in the past two decades.

## 2 METHODS

We performed a bibliometric analysis on January 1st, 2021 by using the Web of Science (WoS) database using the search terms “Stroke” and “Neurovascular unit” in the title/abstract/keywords between 2001 and 2020. Total citation number and average number of citations for each report of all results were collected in the WoS analysis tool.

Data were extracted from the WoS database to identify collaborations between authors, countries, organizations and keywords within the boundaries of the aforementioned search terms using VOSviewer (1.6.16 mac). Co‐authorship analysis and co‐occurrence analysis were performed, and the number of authors, countries, organizations, and keywords was based on the choice of a minimum number of documents or occurrences. Two bibliometric indicators, the activity index (AI) and category normalized citation impact (CNCI), were computed. AI indicates the relative research effort of a country to a research field based on publications. The CNCI of an article is the ratio of the observed number of citations to the expected number of citations. If AI or CNCI was greater than 1, then the research power or the academic influence of a country was higher than the global average, and vice versa.

The initial results were then filtered in descending order of the number of citations in WoS to find the 100 most‐cited articles. The article title, number of citations, publication year, journal, journal impact factor (IF), first author, corresponding author, country/institution of the corresponding author, and study type were collected. Eight articles were excluded after reviewing the title, abstract, and whole text of these papers by two experienced anesthesiologists in our group. Study type was accessed by the two doctors mentioned above reading the full text. These data were then imported to Microsoft Excel for analysis manually. The keywords of bursts were also identified by CiteSpace.

## 3 SEARCH RESULTS

We found 774 articles in the initial search of the WoS database under the search terms “Stroke” and “Neurovascular unit.” Of these papers, 20 176 reports had cited these articles, with a total citation number of 28141. The average number of citations for each report was 36.26.

**FIGURE 1:**
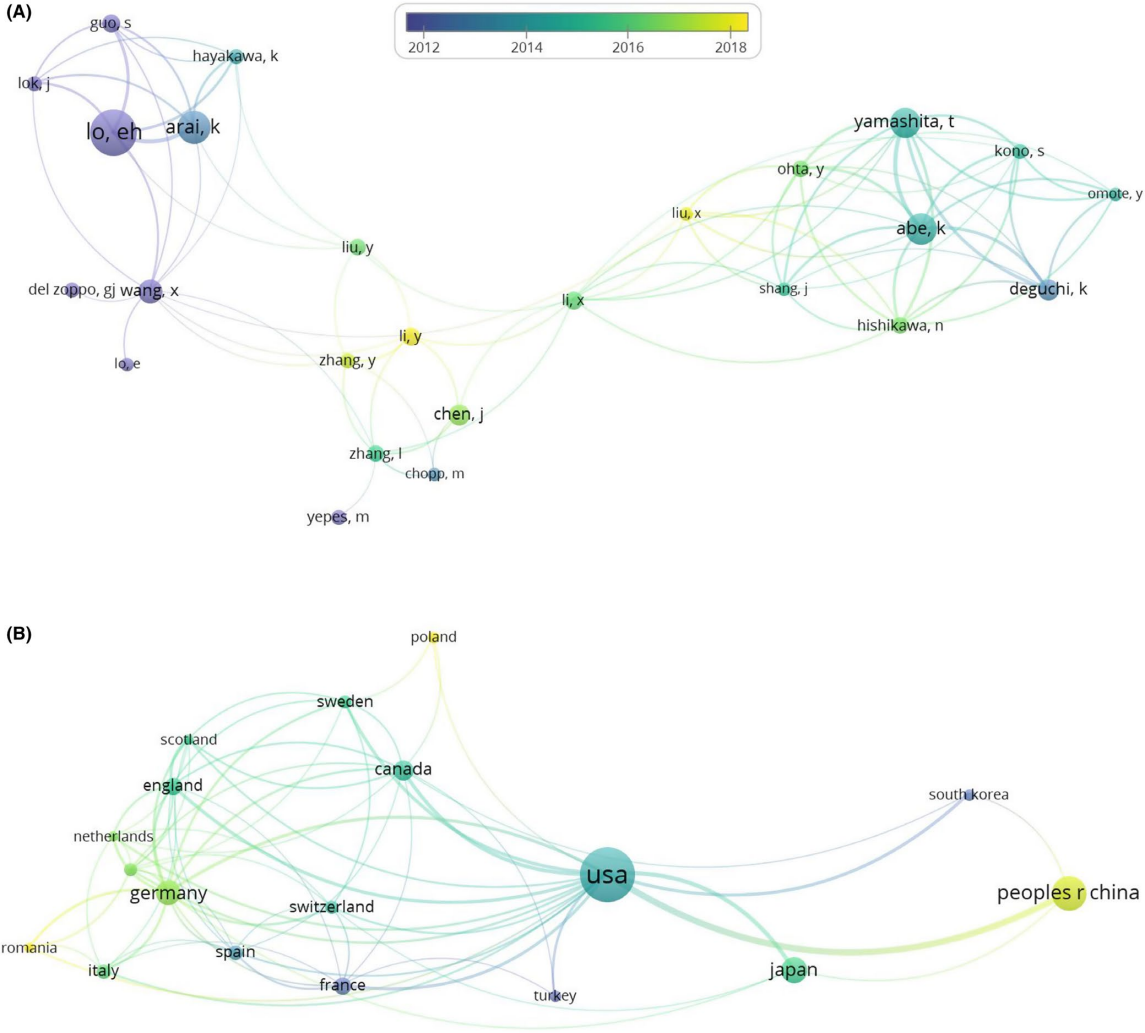
VOSviewer analysis of collaborations between authors and countries based on the co‐authorship analysis. (A) Two different clusters of authors were identified which represented American and Japanese authors. A total of 2,662 authors were counted, and we set the minimum number of documents of an author for 9 and 27 authors meet the threshold. 25 authors were shown as 2 authors were not connected to each other. (B) Three different clusters of countries were identified which represented USA, European, and East Asian countries. A total of 50 countries were counted, and we set the minimum number of documents of a country for 8 and 18 countries meet the threshold. The size of the circle is weighed by documents of the author and country. The thickness of the connecting lines reflects the strength of collaborations. The color of each circle represents the order of time, the more yellow the color is, the closer the time, and purple farther

Seven‐hundred and seventy articles were analyzed by VOSviewer after excluding one withdrawn article and three patent documents. The result of co‐authorship analysis, which includes 27 authors with >9 reports, is shown in Figure 1A. Among the 27 authors, two of them were not connected to each other. Two different clusters of authors were identified, which represented American and Japanese authors. The result of country co‐authorship analysis is shown in Figure 1B. A minimum of 8 publications per country was required for the country to be included in the analysis. Three different clusters of countries were identified, which represented the USA, European, and East Asian countries. We also performed organization co‐authorship analysis and keywords co‐occurrence analysis. Nineteen organizations and 20 keywords that met the threshold are shown in Figure S1. The size of the circle is weighted by documents or occurrences of the author, country, organization, and keyword. The thickness of the connecting lines reflects the strength of collaborations. The color of each circle represents the order of time, the more yellow the color is the closer the time, whereas purple is the farthest in time.

**FIGURE 2:**
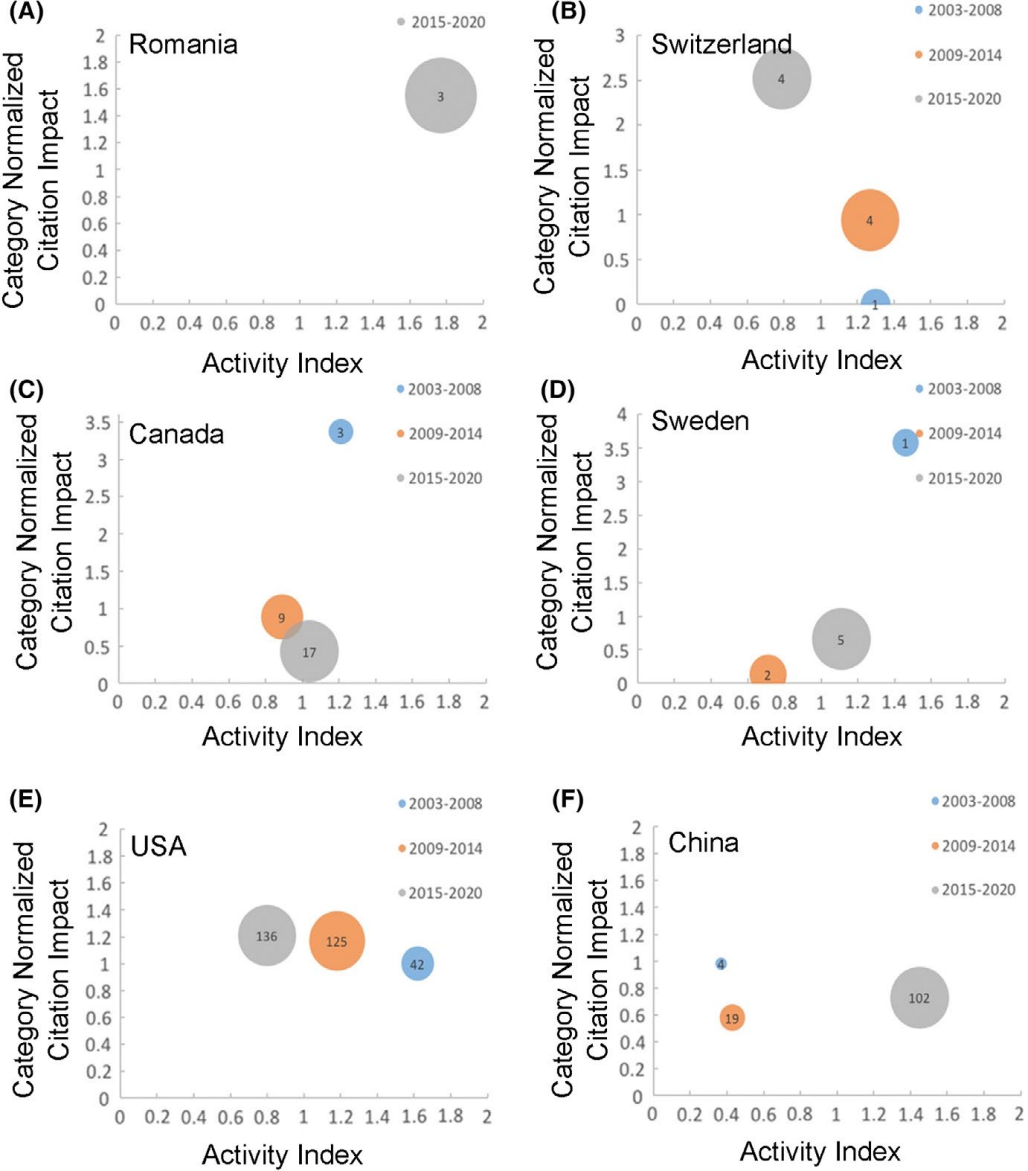
Changes in Activity Index and Category Normalized Citation Impact for the periods 2003–2008, 2009–2014 and 2015–2020 for the 6 countries in the research of stroke and neurovascular unit. (A) Romania has emerged as new‐star country. (B) Switzerland increased in CNCI, but decreased in AI. (C, D) Canada and Sweden had a sharp decline in influence in CNCI. (E) USA had steady improvement in CNCI but decreased in AI. (F) China shown great increase in AI which indicates research effort and the number of articles from China increased sharply from 2003–2008 to 2015–2020. If AI or CNCI was greater than 1, then the research power or the academic influence of a country was higher than the global average, and vice versa. The size of the bubbles represented the number of articles one country published in the time period

We computed AIs and CNCIs of the eighteen countries according to VOSviewer co‐authorship analysis results for the periods 2003–2008, 2009–2014, and 2015–2020. At the country level, AIs and CNCIs varied across study periods and from one country to another (Table 1). To interpret the trends better, AIs were plotted against CNCIs for each of the eighteen countries for the periods 2003–2008, 2009–2014, and 2015–2020 (Figure 2, Figure S2). The size of the bubbles represents the number of articles one country published. Romania, Hungary, and Poland have emerged as rising‐star countries. Switzerland increased in CNCI, but decreased in AI. Canada, Sweden, Turkey, and Australia had a sharp decline in influence in CNCI. The USA had steady improvement in CNCI but decreased in AI. China showed great increase in AI, which indicates research effort, and the number of articles from China increased sharply from 2003–2008 to 2015–2020. France declined in both AI and CNCI. Spain and South Korea had a sharp decline in AI. Other countries showed relatively little change.

**TABLE 1:** AIs and CNCIs for the 18 Countries for three 6‐year time periods, listed in descending order of the total number of citations

	Total	2003–2008			2009–2014			2015–2020		
		*N*	SI	CNCI	*N*	SI	CNCI	*N*	SI	CNCI
World	770	66			270			434		
USA	303	42	1.62	1.00	125	1.18	1.17	136	0.80	1.21
China	125	4	0.37	0.98	19	0.43	0.58	102	1.45	0.73
Japan	59	0	0.00	0.00	28	1.35	0.45	31	0.93	0.51
Germany	52	0	0.00	0.00	17	0.93	0.99	35	1.19	0.85
France	38	4	1.23	0.35	23	1.73	1.13	11	0.51	0.16
Canada	29	3	1.21	3.37	9	0.89	0.89	17	1.04	0.43
England	21	2	1.11	0.87	8	1.09	1.38	11	0.93	0.82
Spain	18	3	1.94	0.35	9	1.43	0.57	6	0.59	0.69
Italy	14	0	0.00	0.00	5	1.02	0.41	9	1.14	0.51
Australia	10	0	0.00	0.00	4	1.14	2.24	6	1.06	0.98
Turkey	9	1	1.30	1.61	3	0.95	0.84	5	0.99	0.54
Switzerland	9	1	1.30	0.00	4	1.27	0.94	4	0.79	2.52
Poland	9	0	0.00	0.00	2	0.63	0.49	7	1.38	1.26
Sweden	8	1	1.46	3.58	2	0.71	0.13	5	1.11	0.66
South Korea	7	2	3.33	0.44	2	0.81	0.79	3	0.76	0.70
Hungary	6	0	0.00	0.00	1	0.48	0.01	5	1.48	0.54
Romania	3	0	0.00	0.00	0	0.00	0.00	3	1.77	1.55
Scotland	3	0	0.00	0.00	2	1.90	0.38	1	0.59	0.18
Netherlands	1	0	0.00	0.00	0	0.00	0.00	1	1.77	3.80

### 3.1 Characteristics of the 100 most‐cited reports

**FIGURE 3:**
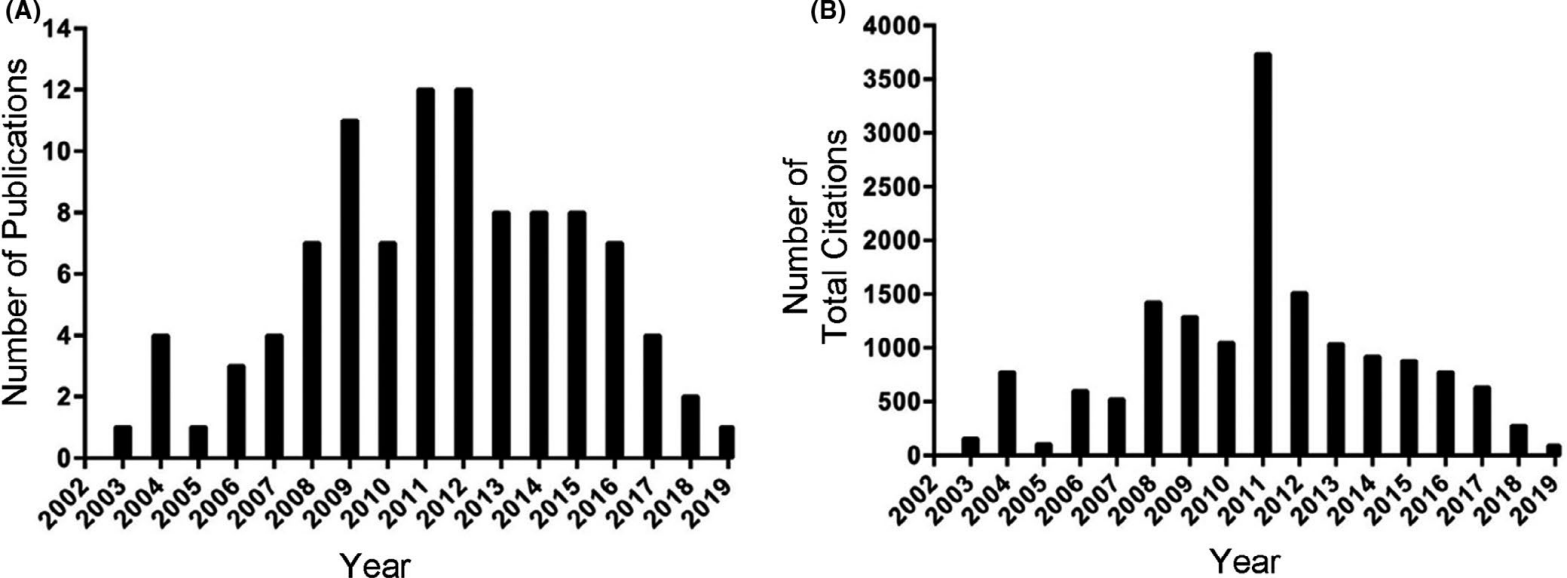
(A) Annual number of publications of the 100 most‐cited articles in Stroke and Neurovascular Unit from 2001 to 2020 in Web of Science. (B) Annual number of total citations of the 100 most‐cited articles in Stroke and Neurovascular Unit

The top 10 most‐cited articles‐reviews and the top 10 most‐cited articles‐basic studies are listed in Tables 2 and 3. Details of the 100 most‐cited articles are listed in descending order of the total number of citations (Table S1). These articles were published between 2003 and 2019. The distribution of the number of publications is shown in Figure 3A. Most articles were published in 2011 and 2012 (*n* = 13 each), followed by 2009 (*n* = 11) and 2013, 2014, and 2015 (*n* = 8 each). In the 100 most‐cited articles, the number of citations ranged from 1873 to 69, with a total of 15758 citations. “Vascular Contributions to Cognitive Impairment and Dementia A Statement for Healthcare Professionals from the American Heart Association/American Stroke Association” published in the journal *Stroke* had 1873 citations, with five articles having >400 citations and eight articles having >200 citations. The trend in the total number of citations by year is shown in Figure 3B. The 100 most‐cited articles were published in 58 journals. Journals that published two or more articles are listed in Table 4. The top journals included *Stroke* with fourteen reports, followed by *Journal of Cerebral Blood Flow and Metabolism* with eleven articles.

**TABLE 2:** The Top‐10 most‐cited articles‐reviews about the role of neurovascular unit in stroke ranked in order of the number of citations

Rank	Year	Article title	Number of citations	First author
1	2011	Vascular contributions to cognitive impairment and dementia a statement for healthcare professionals from the American Heart Association/American Stroke Association	1873	PB Gorelick
2	2008	Blood‐brain barrier tight junction permeability and ischemic stroke	562	KE Sandoval
3	2011	Central nervous system pericytes in health and disease	497	EA Winkler
4	2006	Perivascular nerves and the regulation of cerebrovascular tone	432	E Hamel
5	2011	Blood‐brain barrier breakdown in acute and chronic cerebrovascular disease	421	Y Yang
6	2017	The neurovascular unit coming of age: a journey through neurovascular coupling in health and disease	410	C Iadecola
7	2012	Tight junctions at the blood‐brain barrier: physiological architecture and disease‐associated dysregulation	244	AC Luissint
8	2013	Hemorrhagic transformation after ischemic stroke in animals and humans	227	GC Jickling
9	2004	Mechanisms of hemorrhagic transformation after tissue plasminogen activator reperfusion therapy for ischemic stroke	225	XY Wang
10	2014	The impact of microglial activation on blood‐brain barrier in brain diseases	223	ACC da Fonseca

**TABLE 3:** The Top‐10 most‐cited articles‐basic studies about the role of neurovascular unit in stroke ranked in order of the number of citations

Rank	Year	Article title	Number of citations	First author
1	2008	Activation of PDGF‐CC by tissue plasminogen activator impairs blood‐brain barrier integrity during ischemic stroke	293	EJ Su
2	2004	Reperfusion‐induced oxidative/nitrative injury to neurovascular unit after focal cerebral ischemia	216	Y Gursoy‐Ozdemir
3	2008	Neuroprotection via matrix‐trophic coupling between cerebral endothelial cells and neurons	182	SZ Guo
4	2012	Macrophages prevent hemorrhagic infarct transformation in murine stroke models	163	M Gliem
5	2011	Transplanted stem cell‐secreted vascular endothelial growth factor effects poststroke recovery, inflammation, and vascular repair	156	N Horie
6	2014	MicroRNA‐155 negatively affects blood‐brain barrier function during neuroinflammation	146	MA Lopez‐Ramirez
7	2009	Dissociation and protection of the neurovascular unit after thrombolysis and reperfusion in ischemic rat brain	134	T Yamashita
8	2015	Neuronal interleukin‐4 as a modulator of microglial pathways and ischemic brain damage	131	XR Zhao
9	2012	Paeoniflorin protects against ischemia‐induced brain damages in rats via inhibiting MAPKs/NF‐kappa B‐mediated inflammatory responses	131	RB Guo
10	2013	The neurovascular unit as a selective barrier to polymorphonuclear granulocyte (PMN) infiltration into the brain after ischemic injury	120	Gaby Enzmann

**TABLE 4:** Ranking of journals publishing the 100 most‐cited articles in stroke and neurovascular unit

Rank	Journal	IF	Number of articles
1	*Stroke*	7.19	14
2	*Journal of Cerebral Blood Flow and Metabolism*	5.681	11
3a	*Acta Neuropathologica*	14.256	3
3b	*Innate Inflammation and Stroke*	4.728	3
5a	*British Journal of Pharmacology*	7.73	2
5b	*Current Opinion in Investigational Drugs*	3.553	2
5c	*Current Pharmaceutical design*	2.208	2
5d	*Febs Journal*	4.392	2
5e	*Frontiers in Cellular Neuroscience*	3.921	2
5f	*International Journal of Stroke*	4.882	2
5g	*Journal of Pineal Research*	14.528	2
5h	*Proceedings of the National Academy of Sciences of the United States of America*	9.412	2
5i	*Progress in Neurobiology*	9.371	2
5j	*Stem Cells*	6.002	2
5k	*Trends in Neurosciences*	12.891	2

A total of 90 authors had been listed as the first author. EH Lo (*n* = 3), Y Yang (*n* = 3), K Arai (*n* = 2), J Badaut (*n* = 2), GJ del Zoppo (*n* = 2), U Dirnagl (*n* = 2), SZ Guo (*n* = 2), and XY Wang (*n* = 2) published the greatest number of studies. A total of 74 authors had been listed as the corresponding author. EH Lo (*n* = 9) from Harvard University/Massachusetts General Hospital, GJ del Zoppo (*n* = 3) from Harborview Medical Center/University of Washington Seattle, and GA Rosenberg (*n* = 3) from the University of New Mexico authored the most articles as the corresponding author. As for the geographical distribution, the articles originated from eighteen countries as shown in Table 5. The majority of the publications were from the United States (*n* = 60), followed by France (*n* = 7), Germany (*n* = 6), and China (*n* = 5). A total of 71 affiliated institutions were listed in the reports depending on the corresponding author's affiliation. Harvard University/Massachusetts General Hospital produced fifteen publications, followed by the University of New Mexico with 6 articles and the Henry Ford Hospital with 4 articles.

**TABLE 5:** Countries of origin publishing the 100 most‐cited articles in stroke and neurovascular unit

Country	Number of articles
USA	60
France	7
Germany	6
China	5
England	4
Australia	2
Canada	2
Colombia	2
Japan	2
Turkey	2
Austria	1
Brazil	1
Finland	1
Ireland	1
Netherlands	1
Spain	1
Sweden	1
Switzerland	1

In terms of the publication form, the articles could be divided into the following categories (Table 6): review (*n* = 70, ten of which were proceeding papers), basic study (*n* = 27), clinical trial (*n* = 1), book chapter (*n* = 1), and editorial material (*n* = 1). In the 70 reviews, seven articles discussed the topic of blood‐brain barrier (BBB), followed by tPA with six articles, pericytes with five articles, angiogenesis/neurovascular coupling with four articles, Alzheimer's disease/astrocyte/matrix metalloproteinase (MMP) with three articles each, and up to 25 articles mentioning therapeutic strategies. In the 27 basic studies, four were about the BBB, followed by stem cell with three publications, and melatonin/MMP/pericytes with 2 articles each. Fourteen articles tried to find therapeutic strategies for stroke. The clinical trial “Reparative Therapy for Acute Ischemic Stroke with Allogeneic Mesenchymal Stem Cells from Adipose Tissue: A Safety Assessment A Phase II Randomized, Double‐blind, Placebo‐controlled, Single‐center, Pilot Clinical Trial” was just a protocol.

**TABLE 6:** Study types of the 100 most‐cited articles in stroke and neurovascular unit

Study type	Number of articles
Review	70
Basic study	27
Book chapter	1
Editorial material	1
Clinical trial	1

**FIGURE 4:**
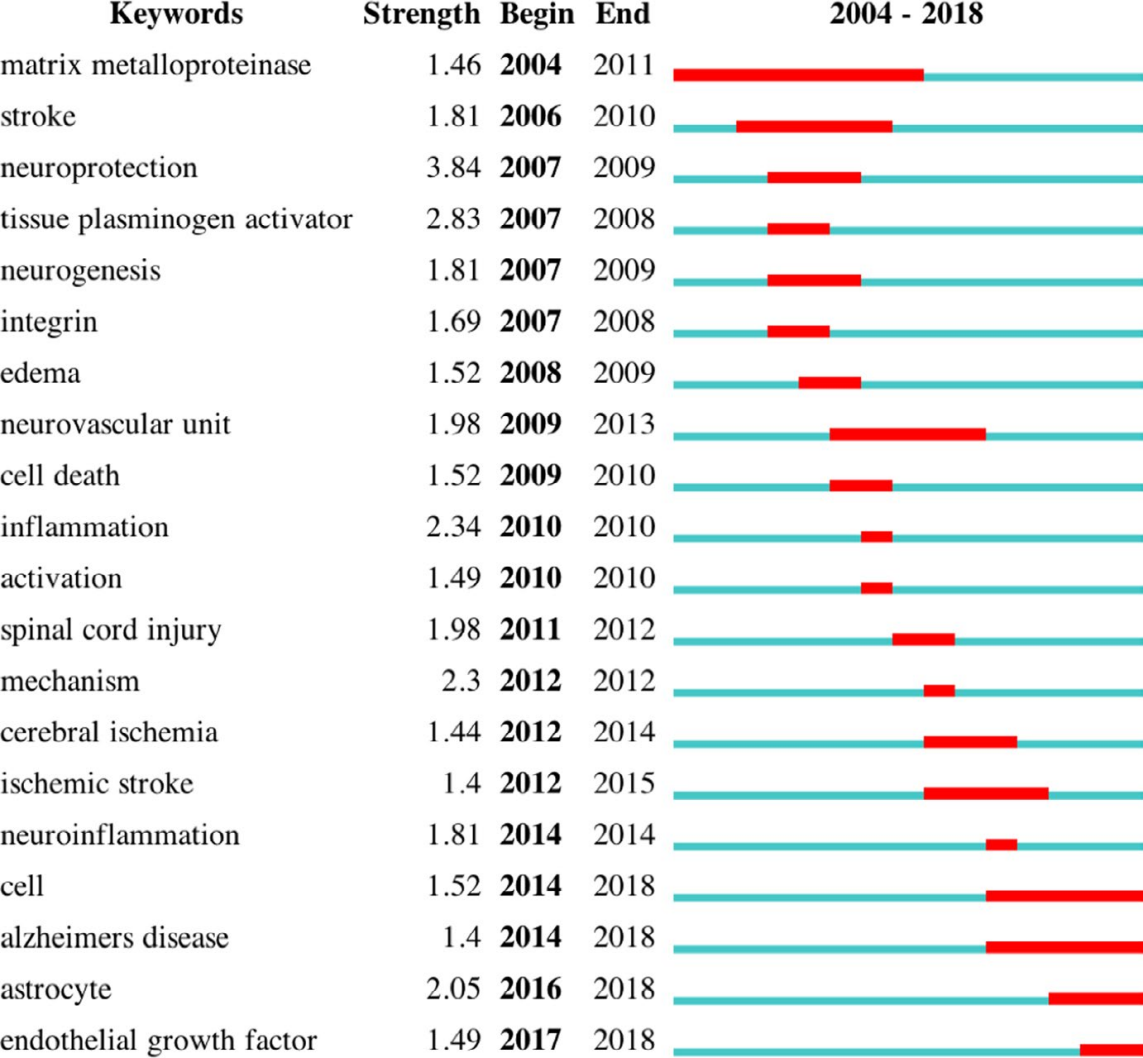
CiteSpace bursts detection was used to capture burst keywords. A total of 20 keywords with strong citation burst in the 100 most‐cited articles published about the role of neurovascular unit in stroke, together with their strength and duration. The strength value reflected the frequency of citation. The green blocks represented the year 2004–2018. The red blocks represented the begin, the end, and the duration of citation bursts

CiteSpace was used to capture burst keywords, the indicators of frontier topics over time. A total of 20 bursts keywords were obtained, together with their strength and the duration, as shown in Figure 4.

## 4 DISCUSSION

The neurovascular unit had been proposed in 2001 as a set of cells and structures, including neurons, pericytes, astrocytes, and microglia that should be investigated in an integrated context. Herein, we analyzed the time period between 2001 and 2020. Most articles were published between 2009 and 2012, about a decade after the concept of the “neurovascular unit” was proposed. Given the fact that citations usually need 3–10 years to accumulate after publication,^25^ the trend in the total number of citations by year peaked in 2011 with a total number of 3,736 citations, as the most‐cited article with 1,873 citations was published in 2011.

VOSviewer showed a visualized view of the relationship between each item. In this study, EH Lo was foremost among American authors, while K Abe was foremost among Japanese researchers. These two clusters are connected by some Chinese and Japanese researchers. This overlap can be explained by overseas post‐doctoral researchers or further education program scholars returning to their home countries and continuing to collaborate within the framework of international networks, thereby increasing the connections between countries. Besides, the USA maintains a dominant position worldwide and has strong connection with China, Japan, Canada, Sweden, and England. These countries also have relatively large bubbles, which are in accordance with the result of the AIs and CICNs analyses. There are also close ties among European countries. Trends in collaborations between authors and countries, which can be illustrated by VOSviewer, are required to highlight changes over time.

AIs and CNCIs analyses provide different perspectives and allow identification of different trends in research output. We found that there was an overall increase in research output. Romania, Hungary, and Poland have emerged as rising‐star countries but with only a few papers. CNCI is an average value, which is affected by sample size and baseline value. Romania, Hungary, and Poland had highly cited papers in recent years, which could have had a huge impact on their CNCI. The USA showed steady improvement in the CNCI, which indicates its great influence in this field but had a decrease in AI. China showed a great increase in research effort, and the number of articles from China increased sharply from 2003–2008 to 2015–2020; however, little change has been seen in its CNCI. Meanwhile, VOSviewer co‐author country analysis of China suggests a relatively big bubble, but VOSviewer co‐author analysis only showed American and Japanese authors with articles >9, which indicates that researchers in China might be scattered and that influential authors still need time to repatriate. AIs and CNCIs combined with VOSviewer could give readers a more in depth understanding of current development of the field.

The article “Neuroinflammation: friend and foe for ischemic stroke” written by RL Jayaraj, and corresponded by Rosenberg GA in 2019, emerged as a “Hot Paper,” which is defined as ranking among the top 0.1% of papers in the academic field of clinical medicine in WoS. In this article,^26^ the authors proposed that neuroinflammation had both beneficial and detrimental roles in stroke, while the NVU, including microglia, astrocytes, endothelial cells, BBB, and leukocytes participated in the pathogenesis of stroke. Understanding the temporal dynamics of immune cells during stroke and the involvement of the pathological mediators, such as oxidative stress, excitotoxicity, matrix metalloproteinases (MMPs), transcriptional modifications, mitogen‐activated protein kinase (MAPK), and high‐mobility group box protein family (HMGB), might provide time‐defined diagnostic, prognostic, and therapeutic neuroprotective strategies and ideas for future studies. This hot paper led to a deeper understanding of the close relationship between the NVU and neuroinflammation, and proposed divergent roles of immune responses on stroke outcome. Five papers had >400 citations, which is defined as “citation classics.” These papers are of high quality and have had wide and significant impact on subsequent stroke research.

*Stroke* with an IF of 7.19 and the *Journal of Cerebral Blood Flow and Metabolism* with 5.681 are two top journals in this field. Researchers were likely to contribute their articles to more influential journals in one field. Thus, we searched the latest papers in these two journals for progress in stroke and the NVU. One paper suggested that caveolin‐1 might have a potential protective role in neovascularization, astrogliosis, and scar formation.^27^ Meanwhile, nitroxide radical‐containing nanoparticles (RNPs) could preserve the endothelial tight junctions and BBB integrity, improving oxygen species scavenging capacity.^28^ The total citation number of “stroke” and “neurovascular unit” related publications in these two journals adds up to 5161 between 2004 and 2016.

Since the majority of the publications related to “stroke” and “neurovascular unit” were from the United States, we also analyzed the specialties of these authors. We found that most of the authors were also prominent in the field of traumatic brain injury,^29^ vascular cognitive impairment,^30^, ^31^ and neuroimaging,^32^, ^33^ suggesting that the NVU is a field that is getting multidisciplinary attention.

In terms of the publication types, most of the 100 most‐cited articles were reviews, and one‐third were basic studies. There was increasing research effort into mechanisms underlying the role the NVU plays after stroke in the pursuit of novel therapeutic strategies for stroke patients. In these 29 basic studies, components of the NVU such as the BBB, tight junction, pericytes, astrocyte, endothelial cells, macrophages, and microglia were widely discussed. Stem cell therapy was mentioned in three articles. Hemorrhagic transformation, oxidative/reperfusion injury, and tPA were still hot topics. Potential therapeutic drugs and targets including melatonin, adenosine, curcumin, paeoniflorin (PF), pinocembrin, tissue inhibitor of MMP, bioactive lipids, stromal cell, polymorphonuclear granulocytes (PMN), (4‐phenoxyphenylsulfinyl) methylthiirane (SB‐3CT), purinergic receptor (P2RY12), brain‐derived neurotrophic factor (BDNF), platelet‐derived growth factor (PDGF), 12/15 lipoxygenase (12/15‐LOX), Rho kinase, sphingosine kinase (SphK), IL‐4, cascade‐3, microRNA, and PPAR‐γ were also investigated in these basic studies, providing pre‐clinical insights into the development of potential therapeutic strategies.

Although many of the above treatments have proved effective in animal stroke models, none have proved effective in treating stroke patients in clinical trials until now,^34^ perhaps due to differences between animal models and humans. Meanwhile, stroke patients often have comorbid disease, such as hypertension, diabetes, hyperlipemia, and even aging,^2^, ^14^, ^35^, ^36^ which might impact the effectiveness of the therapy. Accurate animal models that better match with the stroke population should be established and the role of NVU in stroke requires further investigation. The above findings may help to shape research policy both at the country and institutional level.

### 4.1 Limitations

One of the major limitations of the current study is that we used only “stroke” and “neurovascular unit” as search terms; thus, a lot of studies that did not use the NVU term may have been missed. Another limitation is that given the fact that citations gradually reach a peak in 3–10 years after publication, the current analysis could not underscore the recently published articles. Our study had small sample size, which could have huge impact on CNCI.

## CONFLICT OF INTEREST

We declare there is no conflict of interest among all the authors.

## Supporting information

App S1

## Data Availability

The data that support the findings of this study are available on request from the corresponding author.
