# Supplementary material for: The 100 most‐cited articles about the role of neurovascular unit in stroke 2001–2020: A bibliometric analysis
Source: CNS Neurosci Ther. 2021 Mar 25;27(7):743–52. doi: 10.1111/cns.13636 (PMC8193691; doi:10.1111/cns.13636)
Supplement: Supplementary file 1 — App S1 [file CNS-27-743-s001.docx]

**Supplementary Materials**

**The 100-most Cited Articles About Stroke and Neurovascular Unit 2001-2020: A Bibliometric Analysis**

Lv Xie^1#^, Bingwei Lu^1#^, Yezhi Ma^1^, Jiemin Yin^1^, Xiaozhu Zhai^1^, Chen Chen^1^, Wanqin Xie^1^, Yueman Zhang^1^, Li Zheng^1*^, Peiying Li^1*^

^1^ Department of Anesthesiology, State Key Laboratory of Oncogenes and Related Genes, Shanghai Cancer Institute, Renji Hospital, School of Medicine Shanghai Jiaotong University, Shanghai 200127, China

Running Title: Stroke and Neurovascular Unit

Supplementary Table 1. The 100 Most-Cited Articles in Stroke and Neurovascular Unit Ranked in Order of Citation Numbers

| Rank | Year | Article Title | Number of Citations | Number of Annual Citations |
| --- | --- | --- | --- | --- |
| 1 | 2011 | Vascular Contributions to Cognitive Impairment and Dementia A Statement for Healthcare Professionals From the American Heart Association/American Stroke Association | 1873 | 208.11 |
| 2 | 2008 | Blood-brain barrier tight junction permeability and ischemic stroke | 562 | 46.83 |
| 3 | 2011 | Central nervous system pericytes in health and disease | 497 | 55.22 |
| 4 | 2006 | Perivascular nerves and the regulation of cerebrovascular tone | 432 | 30.86 |
| 5 | 2011 | Blood-Brain Barrier Breakdown in Acute and Chronic Cerebrovascular Disease | 421 | 46.78 |
| 6 | 2017 | The Neurovascular Unit Coming of Age: A Journey through Neurovascular Coupling in Health and Disease | 410 | 136.67 |
| 7 | 2008 | Activation of PDGF-CC by tissue plasminogen activator impairs blood-brain barrier integrity during ischemic stroke | 293 | 24.42 |
| 8 | 2012 | Tight junctions at the blood brain barrier: physiological architecture and disease-associated dysregulation | 244 | 30.50 |
| 9 | 2013 | Hemorrhagic transformation after ischemic stroke in animals and humans | 227 | 32.43 |
| 10 | 2004 | Mechanisms of hemorrhagic transformation after tissue plasminogen activator reperfusion therapy for ischemic stroke | 225 | 14.06 |
| 11 | 2014 | The impact of microglial activation on blood-brain barrier in brain diseases | 223 | 37.17 |
| 12 | 2004 | Reperfusion-induced oxidative/nitrative injury to neurovascular unit after focal cerebral ischemia | 216 | 13.50 |
| 13 | 2010 | Angiogenesis, neurogenesis and brain recovery of function following injury | 212 | 21.20 |
| 14 | 2009 | Tissue-type plasminogen activator in the ischemic brain: more than a thrombolytic | 207 | 18.82 |
| 15 | 2013 | Neuronal oxidative stress in acute ischemic stroke: Sources and contribution to cell injury | 195 | 27.86 |
| 16 | 2010 | NF-kappa B and innate immunity in ischemic stroke | 191 | 19.10 |
| 17 | 2010 | Molecular insights and therapeutic targets for blood-brain barrier disruption in ischemic stroke: Critical role of matrix metalloproteinases and tissue-type plasminogen activator | 188 | 18.80 |
| 18 | 2009 | Brain angiogenesis in developmental and pathological processes: neurovascular injury and angiogenic recovery after stroke | 187 | 17.00 |
| 19 | 2007 | Cell-cell signaling in the neurovascular unit | 182 | 14.00 |
| 20 | 2008 | Neuroprotection via matrix-trophic coupling between cerebral endothelial cells and neurons | 182 | 15.17 |
| 21 | 2009 | Injury and repair mechanisms in ischemic stroke: Considerations for the development of novel neurotherapeutics | 181 | 16.45 |
| 22 | 2016 | Astrocytes, therapeutic targets for neuroprotection and neurorestoration in ischemic stroke | 179 | 44.75 |
| 23 | 2015 | The dynamic blood-brain barrier | 179 | 35.80 |
| 24 | 2004 | The neurotoxicity of tissue plasminogen activator? | 176 | 11.00 |
| 25 | 2016 | Molecular pathophysiology of cerebral edema | 176 | 44.00 |
| 26 | 2012 | Macrophages prevent hemorrhagic infarct transformation in murine stroke models | 163 | 20.38 |
| 27 | 2012 | Pathophysiologic cascades in ischemic stroke | 157 | 19.63 |
| 28 | 2011 | Transplanted Stem Cell-Secreted Vascular Endothelial Growth Factor Effects Poststroke Recovery, Inflammation, and Vascular Repair | 156 | 17.33 |
| 29 | 2003 | Triggers and mediators of hemorrhagic transformation in cerebral ischemia | 155 | 9.12 |
| 30 | 2004 | TPA and proteolysis in the neurovascular unit | 155 | 9.69 |
| 31 | 2010 | The neurovascular unit in the setting of stroke | 149 | 14.90 |
| 32 | 2014 | MicroRNA-155 negatively affects blood-brain barrier function during neuroinflammation | 146 | 24.33 |
| 33 | 2007 | Neurovascular proteases in brain injury, hemorrhage and remodeling after stroke | 141 | 10.85 |
| 34 | 2018 | Functional morphology of the blood-brain barrier in health and disease | 140 | 70.00 |
| 35 | 2012 | Pathobiology of injury after stroke: the neurovascular unit and beyond | 136 | 17.00 |
| 36 | 2018 | Blood-brain barrier dysfunction and recovery after ischemic stroke | 135 | 67.50 |
| 37 | 2009 | Dissociation and protection of the neurovascular unit after thrombolysis and reperfusion in ischemic rat brain | 134 | 12.18 |
| 38 | 2015 | Neuronal Interleukin-4 as a Modulator of Microglial Pathways and Ischemic Brain Damage | 131 | 26.20 |
| 39 | 2012 | Paeoniflorin Protects against Ischemia-Induced Brain Damages in Rats via Inhibiting MAPKs/NF-kappa B-Mediated Inflammatory Responses | 131 | 16.38 |
| 40 | 2010 | Mast cells as early responders in the regulation of acute blood-brain barrier changes after cerebral ischemia and hemorrhage | 130 | 13.00 |
| 41 | 2011 | Targeting the Nrf2-Keap1 antioxidant defence pathway for neurovascular protection in stroke | 129 | 14.33 |
| 42 | 2014 | Aquaporin and brain diseases | 128 | 21.33 |
| 43 | 2012 | The vascular neural network-a new paradigm in stroke pathophysiology | 128 | 16.00 |
| 44 | 2008 | Pharmacological approaches to acute ischaemic stroke: reperfusion certainly, neuroprotection possibly | 127 | 10.58 |
| 45 | 2013 | The neurovascular unit as a selective barrier to polymorphonuclear granulocyte (PMN) infiltration into the brain after ischemic injury | 120 | 17.14 |
| 46 | 2012 | Sulfonylurea receptor 1 in central nervous system injury: a focused review | 119 | 14.88 |
| 47 | 2011 | Brain microvascular pericytes in health and disease | 119 | 13.22 |
| 48 | 2013 | Evidence of endothelial dysfunction in the development of Alzheimer's disease: Is Alzheimer's a vascular disorder? | 118 | 16.86 |
| 49 | 2011 | Molecular pathogenesis of blood-brain barrier breakdown in acute brain injury | 115 | 12.78 |
| 50 | 2014 | Squalenoyl adenosine nanoparticles provide neuroprotection after stroke and spinal cord injury | 113 | 18.83 |
| 51 | 2013 | Early loss of pericytes and perivascular stromal cell-induced scar formation after stroke | 109 | 15.57 |
| 52 | 2015 | Revisiting cerebral postischemic reperfusion injury: new insights in understanding reperfusion failure, hemorrhage, and edema | 107 | 21.40 |
| 53 | 2015 | Blood-brain barrier breakdown involves four distinct stages of vascular damage in various models of experimental focal cerebral ischemia | 106 | 21.20 |
| 54 | 2007 | Intravenous administration of melatonin reduces the intracerebral cellular inflammatory response following transient focal cerebral ischemia in rats | 104 | 8.00 |
| 55 | 2005 | Brain response to injury and neurodegeneration - Endogenous neuroprotective signaling | 103 | 6.87 |
| 56 | 2011 | Cytoprotective protein C pathways and implications for stroke and neurological disorders | 103 | 11.44 |
| 57 | 2013 | A Novel Brain Neurovascular Unit Model with Neurons, Astrocytes and Microvascular Endothelial Cells of Rat | 99 | 14.14 |
| 58 | 2009 | Dysfunctional Cell-Cell Signaling in the Neurovascular Unit as a Paradigm for Central Nervous System Disease | 99 | 9.00 |
| 59 | 2008 | Protecting against cerebrovascular injury - Contributions of 12/15-lipoxygenase to edema formation after transient focal ischemia | 99 | 8.25 |
| 60 | 2009 | Pericyte Signaling in the Neurovascular Unit | 98 | 8.91 |
| 61 | 2016 | Immune cell trafficking across the barriers of the central nervous system in multiple sclerosis and stroke | 97 | 24.25 |
| 62 | 2013 | Early inhibition of MMP activity in ischemic rat brain promotes expression of tight junction proteins and angiogenesis during recovery | 96 | 13.71 |
| 63 | 2015 | Angiogenesis-regulating microRNAs and Ischemic Stroke | 96 | 19.20 |
| 64 | 2010 | Biphasic actions of HMGB1 signaling in inflammation and recovery after stroke | 96 | 9.60 |
| 65 | 2007 | Fibrinogen signal transduction as a mediator and therapeutic target in inflammation: Lessons from multiple sclerosis | 94 | 7.23 |
| 66 | 2012 | Stem cell therapy for cerebral ischemia: from basic science to clinical applications | 93 | 11.63 |
| 67 | 2015 | Brain Vascular Pericytes Following Ischemia Have Multipotential Stem Cell Activity to Differentiate Into Neural and Vascular Lineage Cells | 93 | 18.60 |
| 68 | 2012 | Blood-Brain Barrier Integrity and Glial Support: Mechanisms that can be Targeted for Novel Therapeutic Approaches in Stroke | 93 | 11.63 |
| 69 | 2015 | Post-stroke cognitive decline: an update and perspectives for clinical research | 92 | 18.40 |
| 70 | 2019 | Neuroinflammation: friend and foe for ischemic stroke | 91 | 91.00 |
| 71 | 2006 | Advancing the study of stroke in women - Summary and recommendations for future research from an NINDS-sponsored multidisciplinary working group | 89 | 6.36 |
| 72 | 2012 | The neurovascular unit and combination treatment strategies for stroke | 89 | 11.13 |
| 73 | 2016 | Purinergic receptor P2RY12-dependent microglial closure of the injured blood-brain barrier | 89 | 22.25 |
| 74 | 2011 | Aquaporins in Cerebrovascular Disease: A Target for Treatment of Brain Edema? | 86 | 9.56 |
| 75 | 2011 | MMP-Mediated Disruption of Claudin-5 in the Blood-Brain Barrier of Rat Brain After Cerebral Ischemia | 85 | 9.44 |
| 76 | 2014 | Protection after stroke: cellular effectors of neurovascular unit integrity | 84 | 14.00 |
| 77 | 2016 | Exosomes in stroke pathogenesis and therapy | 83 | 20.75 |
| 78 | 2012 | The Role of Pericytes in Blood-Brain Barrier Function and Stroke | 82 | 10.25 |
| 79 | 2017 | The Role of Astrocytes in Neuroprotection after Brain Stroke: Potential in Cell Therapy | 82 | 27.33 |
| 80 | 2008 | Experimental models, neurovascular mechanisms and translational issues in stroke research | 82 | 6.83 |
| 81 | 2009 | Hypoxic Preconditioning-Induced Cerebral Ischemic Tolerance Role of Microvascular Sphingosine Kinase 2 | 81 | 7.36 |
| 82 | 2009 | Mechanisms and targets for angiogenic therapy after stroke | 81 | 7.36 |
| 83 | 2014 | The Immune Response to Acute Focal Cerebral Ischemia and Associated Post-stroke Immunodepression: A Focused Review | 79 | 13.17 |
| 84 | 2006 | Melatonin decreases neurovascular oxidative/nitrosative damage and protects against early increases in the blood-brain barrier permeability after transient focal cerebral ischemia in mice | 79 | 5.64 |
| 85 | 2010 | The neurovascular unit, matrix proteases, and innate inflammation | 79 | 7.90 |
| 86 | 2011 | Cellular Mechanisms of Neurovascular Damage and Repair After Stroke | 79 | 8.78 |
| 87 | 2008 | Responses of endothelial cell and astrocyte matrix-integrin receptors to ischemia mimic those observed in the neurovascular unit | 78 | 6.50 |
| 88 | 2012 | t-PA-specific modulation of a human blood-brain barrier model involves plasmin-mediated activation of the Rho kinase pathway in astrocytes | 78 | 9.75 |
| 89 | 2014 | Reparative Therapy for Acute Ischemic Stroke with Allogeneic Mesenchymal Stem Cells from Adipose Tissue: A Safety Assessment A Phase II Randomized, Double-blind, Placebo-controlled, Single-center, Pilot Clinical Trial | 77 | 12.83 |
| 90 | 2009 | The Neurovascular Unit in Health and Disease Introduction | 77 | 7.00 |
| 91 | 2015 | Formation and maintenance of the BBB | 76 | 15.20 |
| 92 | 2009 | Neuroprotective Effects of Overexpressing Tissue Inhibitor of Metalloproteinase TIMP-1 | 76 | 6.91 |
| 93 | 2016 | Curcumin-loaded embryonic stem cell exosomes restored neurovascular unit following ischemia-reperfusion injury | 75 | 18.75 |
| 94 | 2011 | Impact of tissue plasminogen activator on the neurovascular unit: from clinical data to experimental evidence | 73 | 8.11 |
| 95 | 2016 | The Science of Vascular Contributions to Cognitive Impairment and Dementia (VCID): A Framework for Advancing Research Priorities in the Cerebrovascular Biology of Cognitive Decline | 72 | 18.00 |
| 96 | 2013 | A Concerted Appeal for International Cooperation in Preclinical Stroke Research | 70 | 10.00 |
| 97 | 2017 | Dysfunction of the neurovascular unit in ischemic stroke and neurodegenerative diseases: An aging effect | 69 | 23.00 |
| 98 | 2017 | Pinocembrin protects hemorrhagic brain primarily by inhibiting toll-like receptor 4 and reducing M1 phenotype microglia | 69 | 23.00 |
| 99 | 2014 | Emerging Roles of Pericytes in the Regulation of the Neurovascular Unit in Health and Disease | 69 | 11.50 |
| 100 | 2009 | Cell-derived microparticles: a new challenge in neuroscience | 69 | 6.27 |


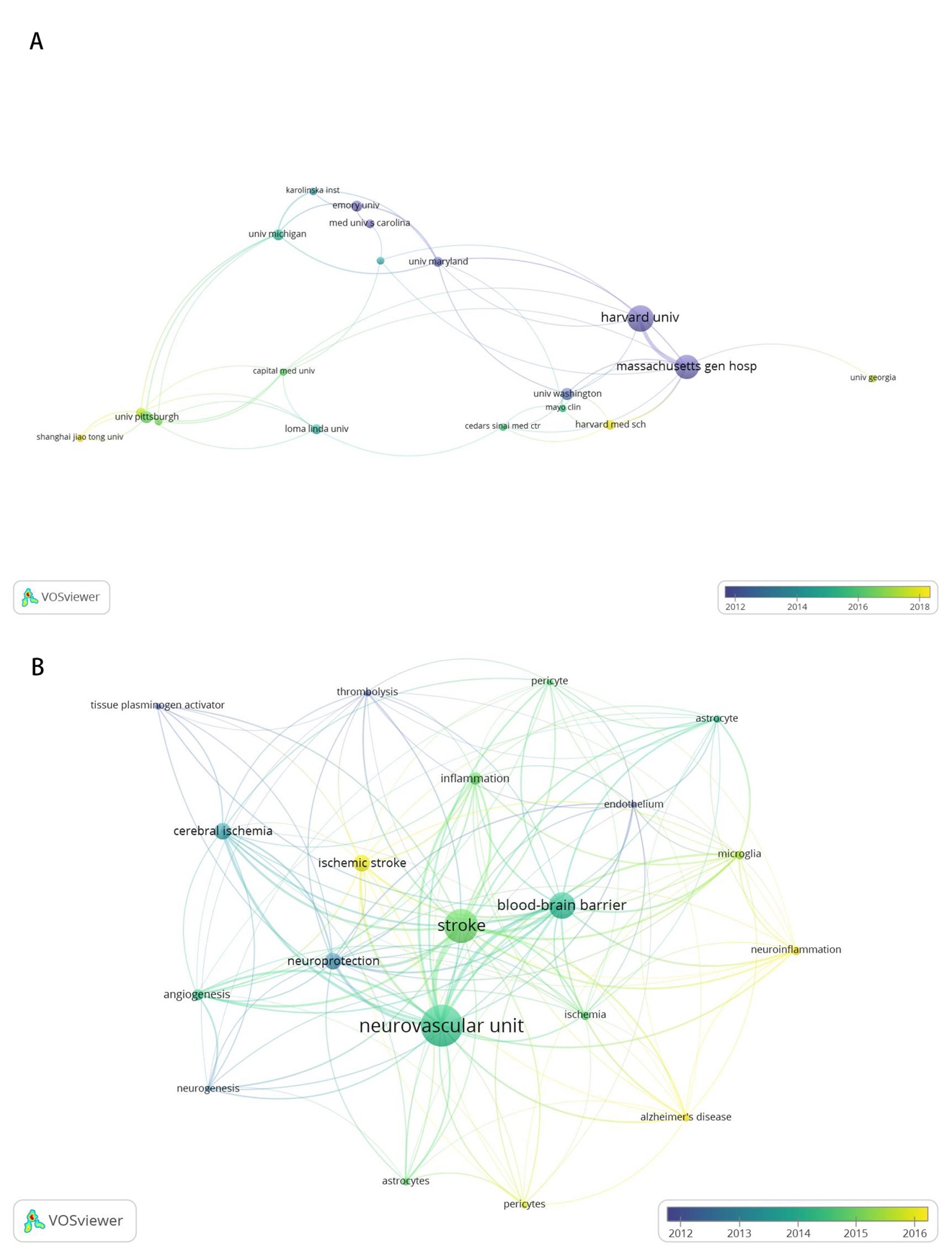


Supplementary Figure 1. VOSviewer analysis of collaborations between institutions and keywords based on the co-authorship and co-occurrence analysis. (A) A total of 748 organizations were counted, we set the minimum number of documents of an organization for 8 and 22 meet the threshold. 19 organizations were shown as 3 organizations were not connected to each other. (B) A total of 1471 author keywords were counted, we set the minimum number of occurrences for 16 and 20 keywords meet the threshold. The size of the circle is weighed by documents and occurrences of the organization and keyword. The thickness of the connecting lines reflects the strength of collaborations. The color of each circle represents the order of time, the more yellow the color is, the closer the time, and purple farther.


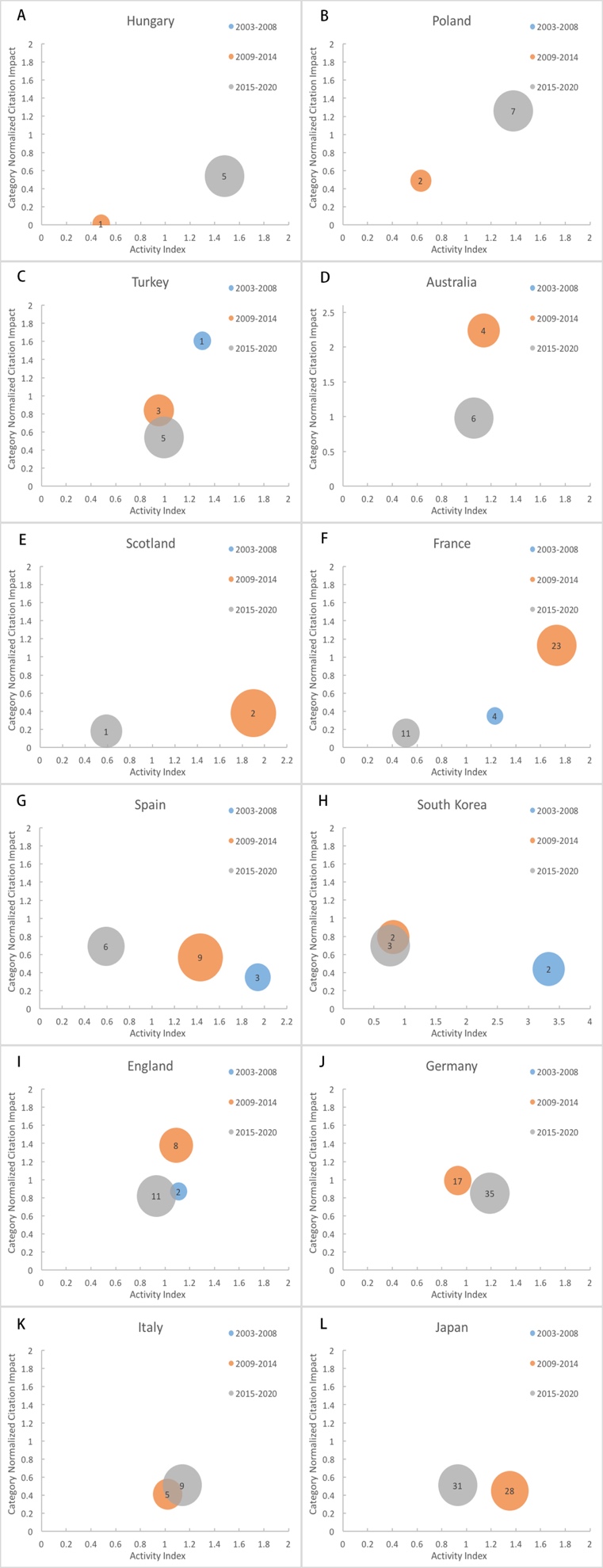


Supplementary Figure 2. Changes in Activity Index and Category Normalized Citation Impact for the periods 2003–2008, 2009-2014 and 2015–2020 for the 12 countries in Stroke and Neurovascular Unit. (A, B) Hungary and Poland have emerged as new-star countries. (C, D) Turkey and Australia declined sharply in CNCI. (E) Scotland decreased in AI. (F) France had a turning point of decline in both AI and CNCI. (G, H) Spain and South Korea had a sharp decline in AI. (I, J, K, L) Other countries showed relatively little change. If AI or CNCI was greater than 1, then the research power or the academic influence of a country was higher than the global average, and vice versa. The size of the bubbles represented the number of articles one country published in the time period.
